# Rapamycin Combined with Anti-CD45RB mAb and IL-10 or with G-CSF Induces Tolerance in a Stringent Mouse Model of Islet Transplantation

**DOI:** 10.1371/journal.pone.0028434

**Published:** 2011-12-09

**Authors:** Nicola Gagliani, Silvia Gregori, Tatiana Jofra, Andrea Valle, Angela Stabilini, David M. Rothstein, Mark Atkinson, Maria Grazia Roncarolo, Manuela Battaglia

**Affiliations:** 1 San Raffaele Diabetes Research Institute, San Raffaele Scientific Institute, Milan, Italy; 2 San Raffaele Telethon Institute for Gene Therapy, San Raffaele Scientific Institute, Milan, Italy; 3 Vita-Salute San Raffaele University, Milan, Italy; 4 Starzl Transplantation Institute, University of Pittsburgh Medical Center, Pittsburgh, Pennsylvania, United States of America; 5 Department of Pathology, The University of Florida, Gainesville, Florida, United States of America; University of Colorado Denver, United States of America

## Abstract

**Background:**

A large pool of preexisting alloreactive effector T cells can cause allogeneic graft rejection following transplantation. However, it is possible to induce transplant tolerance by altering the balance between effector and regulatory T (Treg) cells. Among the various Treg-cell types, Foxp3^+^Treg and IL-10–producing T regulatory type 1 (Tr1) cells have frequently been associated with tolerance following transplantation in both mice and humans. Previously, we demonstrated that rapamycin+IL-10 promotes Tr1-cell–associated tolerance in Balb/c mice transplanted with C57BL/6 pancreatic islets. However, this same treatment was unsuccessful in C57BL/6 mice transplanted with Balb/c islets (classified as a stringent transplant model). We accordingly designed a protocol that would be effective in the latter transplant model by simultaneously depleting effector T cells and fostering production of Treg cells. We additionally developed and tested a clinically translatable protocol that used no depleting agent.

**Methodology/Principal Findings:**

Diabetic C57BL/6 mice were transplanted with Balb/c pancreatic islets. Recipient mice transiently treated with anti-CD45RB mAb+rapamycin+IL-10 developed antigen-specific tolerance. During treatment, Foxp3^+^Treg cells were momentarily enriched in the blood, followed by accumulation in the graft and draining lymph node, whereas CD4^+^IL-10^+^IL-4^−^ T (i.e., Tr1) cells localized in the spleen. In long-term tolerant mice, only CD4^+^IL-10^+^IL-4^−^ T cells remained enriched in the spleen and IL-10 was key in the maintenance of tolerance. Alternatively, recipient mice were treated with two compounds routinely used in the clinic (namely, rapamycin and G-CSF); this drug combination promoted tolerance associated with CD4^+^IL-10^+^IL-4^−^ T cells.

**Conclusions/Significance:**

The anti-CD45RB mAb+rapamycin+IL-10 combined protocol promotes a state of tolerance that is IL-10 dependent. Moreover, the combination of rapamycin+G-CSF induces tolerance and such treatment could be readily translatable into the clinic.

## Introduction

T regulatory (Treg) cells typically control immune responses, and they are also capable of establishing tolerance to non-self molecules that are deliberatively introduced into the host, as occurs in allogeneic transplantation settings [1], [2]. However, endogenous Treg cells do not usually occur in sufficient numbers to control the large population of pre-existing alloreactive T effector cells in recipients, and this imbalance increases the potential for graft rejection [3]. Immunosuppressive drugs block/deplete alloreactive T effector cells, and are currently used in the clinic to prevent graft rejection [4]. However, most of these drugs necessitate life-long administration and thus increase the risk of undesirable side effects (*e.g.,* infections and lymphomas). In addition, some immunosuppressive drugs, such as cyclosporine A and FK506, are known to interfere not only with the function of allogeneic T cells but also with that of Treg cells [5]. The twin priorities of overcoming interference with Treg cells and of inducing long-term transplant tolerance argue strongly for a therapeutic approach that simultaneously enables the depletion of pre-existing alloantigen specific T cells and the fostering of Treg cells [1], [6].

The CD4^+^ Treg cells that have most often been associated with tolerance to allogeneic transplantation in mice and humans are CD4^+^CD25^+^Foxp3^+^ (Foxp3^+^) Treg cells and T regulatory type 1 (Tr1) cells. The expression of CD25 is considered essential for the complete fitness of Foxp3^+^ Treg cells [7]. In contrast, Tr1 cells do not constitutively express CD25 and Foxp3, and are defined by the production of high levels of IL-10 and the absence of IL-4, as well as by the predominant occurrence of control immune responses via IL-10 and TGF-β release [8], [9]. On these bases, Foxp3^+^-Treg and Tr1 cells are considered to be two distinct types of Treg cells [10], [11].

We previously established two distinct models of islet transplantation on the basis of the mean rejection time of untreated transplanted mice, whereby one model could be considered as more stringent than the other [12]. Thus differentiated, these two models were used to test different compounds, either alone or in combination, in order to define the optimal protocol for inducing stable long-term tolerance. Moreover, we attempted to design a clinically relevant protocol by restricting our testing to compounds that were already in use within current clinical settings.

Rapamycin is a non-calcineurin-based inhibitor that is currently used in a variety of immunosuppressive strategies, in combination with other compounds, to block solid organ graft rejection [13]. Of note, this drug not only blocks T cell activation, but also selectively allows for proliferation as well as fostering the suppressive function of Foxp3^+^ Treg cells [14], [15]. IL-10 is a cytokine with potent anti-inflammatory properties that can induce Tr1 cells *in vitro* [12], [16]. We previously showed that rapamycin+IL-10 treatment leads to long-term tolerance associated with the induction of Tr1 cells in the non-stringent model of islet transplant [17]. Therefore, a primary goal of this current effort is to test rapamycin+IL-10 therapy in the more stringent mouse model of islet transplantation. Considering the high frequency of alloreactive T cells in the recipient mice of this model, we hypothesized that a depleting agent could improve the efficacy of rapamycin+IL-10 treatment. Therefore, from the several depleting agents available today, we chose to test anti-CD45RB mAb, which transiently depletes alloreactive T cells from the blood [18], [19] and, more importantly, has the capacity to increase the Treg-cell suppressive function [20].

Another promising tolerogenic molecule is the granulocyte-colony stimulating factor (G-CSF), which is currently used in clinical practice for the mobilization of bone marrow hematopoietic stem cells. *In vivo* administration of G-CSF blocks graft versus host disease (GvHD) [21], and prevents type 1 diabetes development [22], [23]. The effect of this molecule in a setting of islet transplantation has never been investigated, and the issue of whether its tolerogenic effect is associated with increases in IL-10 release or with Foxp3^+^ Treg-cell expansion is controversial [21], [22].

The current study demonstrates that, in a stringent model of islet transplantation, the combination of anti-CD45RB mAb with rapamycin+IL-10 promotes a unique state of tolerance IL-10 dependent and associated with two types of Treg cells. Moreover, the combination of rapamycin+G-CSF induces Tr1 cell-associated tolerance. Considering that both rapamycin and G-CSF are currently in clinical use, we believe these data have powerful clinical implications.

## Results

### The combined protocol (anti-CD45RB mAb+rapamycin+IL-10) promotes tolerance in a stringent mouse model of islet transplant

**Figure 1:**
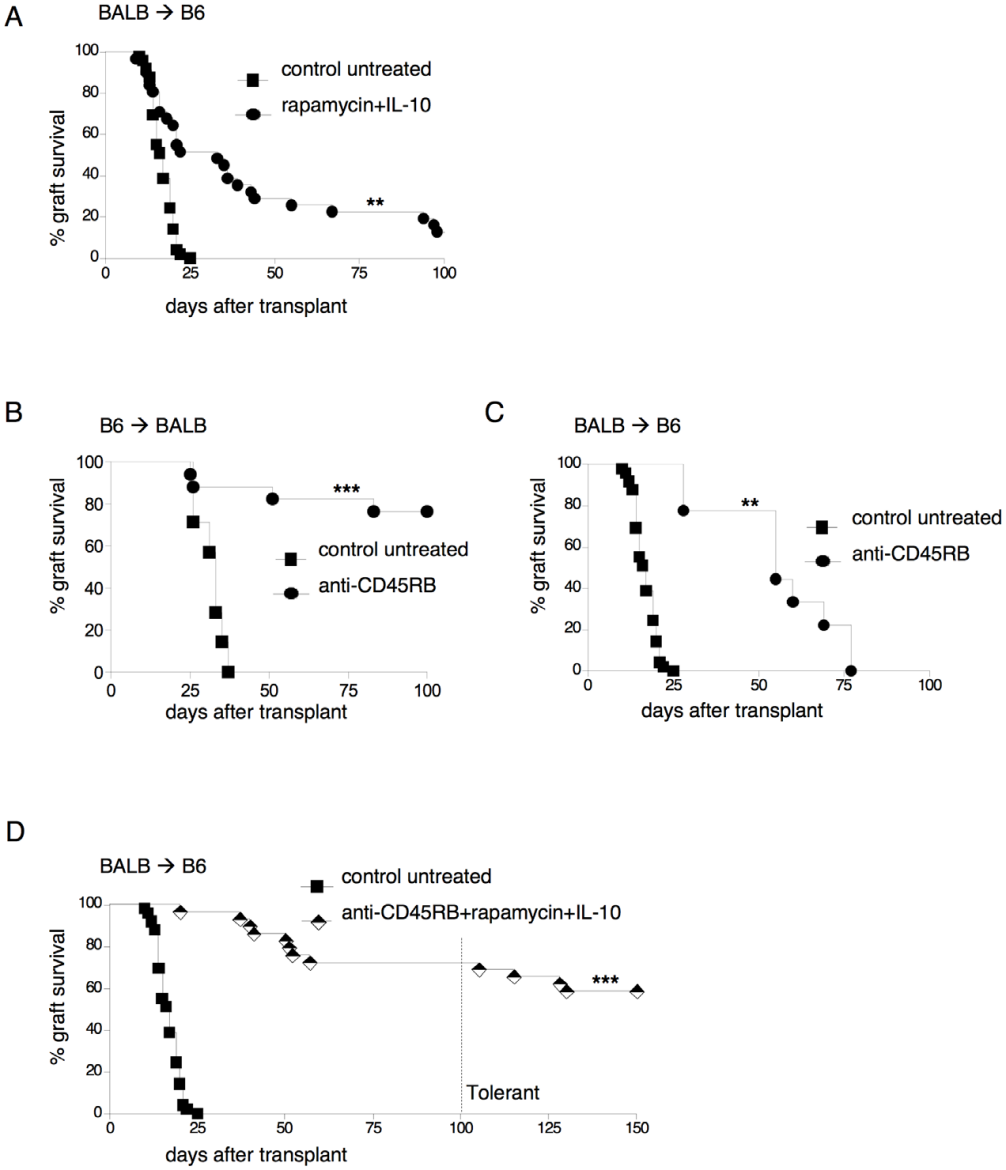
Anti-CD45RB mAb+rapamycin+IL-10 induce long-term tolerance in a stringent model of islet transplantation. (**A**) Diabetic C57BL/6 (B6) mice were transplanted with BALB/c (BALB) islets (BALB→B6). The transplanted mice were either left untreated (n = 49) or treated with rapamycin+IL-10 (n = 30). The treatment was withdrawn 30 days from transplantation. Overall graft survival is shown. (**B**) Diabetic BALB mice were transplanted with B6 islets (B6→BALB). The transplanted mice were either left untreated (n = 17) or treated with anti-CD45RB mAb (n = 17) at days 0, 3 and 5 from transplantation. Overall graft survival is shown. (**C**) Diabetic B6 mice were transplanted with BALB islets (BALB→B6). The transplanted mice were either left untreated (n = 49) or treated with the anti-CD45RB mAb (n = 8) at days 0, 3 and 5 from transplantation. Overall graft survival is shown. (**D**) Diabetic B6 mice were transplanted with BALB islets (BALB→B6). The transplanted mice were either left untreated (n = 49) or treated with anti-CD45RB mAb (at days 0, 3 and 5 from transplantation)+rapamycin+IL-10 (n = 29) for 30 days. One hundred days from transplantation, 30×10^6^ splenocytes isolated from BALB mice were injected in tolerant mice to boost their immune system. Mice that were normoglycemic 40–50 days after the boost were considered tolerant. Overall graft survival is shown. These results are cumulative of 5–10 independent experiments performed in parallel. Log-rank (Mantel-Cox) test has been used (*p<0.01;**p<0.001; ***p<0.0001).

Rapamycin+IL-10 treatment promotes transplantation tolerance in chemically-induced diabetic BALB/c (BALB) mice transplanted with C57BL/6 (B6) islets (B6→BALB) [17]. In a more stringent model of islet transplantation (BALB→B6) rapamycin+IL-10, administered in accordance with a previously used schedule and dose regimen [17], did not promote tolerance (Figure 1A). We hypothesized that a therapy capable of combining deletion with immunomodulant effects would promote long-term tolerance in this more stringent model [1], [6]. Anti-CD45RB mAb transiently depletes circulating T cells [18], which are responsible for allograft rejection [19], [24]; at the same time, it fits the regulatory action of Treg cells [20], [25]. Accordingly, three peri-transplant injections of anti-CD45RB mAb duly promoted long-term acceptance of the graft in 75% of BALB mice transplanted with B6 islets (B6→BALB model) (Figure 1B). This treatment transiently reduced the number of circulating lymphocytes and significantly increased the expression of CTLA-4 on Treg cells in the spleen and in graft-draining lymph nodes (dLN) (data not shown), as previously demonstrated [20].

We then tested the efficacy of anti-CD45RB mAb treatment in the stringent mouse transplantation model (BALB→B6). Although the mAb was highly efficient in blocking early graft rejection (*i.e.,* at 15–30 days following islet transplant), it subsequently lost efficacy (*i.e.,* at 60 days or more following islet transplant) (Figure 1C). On the basis of these data, we designed a combined protocol; to a peri-transplant treatment comprising of anti-CD45RB mAb, we added a 30-day therapy with rapamycin+IL-10. When tested separately, neither rapamycin+IL-10 nor anti-CD45RB mAb attained long-term graft survival in the stringent model of islet transplant (Figure 1A and 1C). Nonetheless, the combined protocol (anti-CD45RB mAb+rapamycin+IL-10) prolonged graft survival in 73% of the mice to 100 days following transplantation (Figure 1D). Significantly, upon injection of donor-derived splenocytes (*i.e.,* a donor-derived antigen boost) 100 days after transplant, only 4 out of 21 mice with a long-term functioning graft rejected the allogeneic islets. Overall, the proportion of mice treated with the combined protocol that achieved long-term tolerance (*i.e.,* acceptance of the graft and maintenance of graft survival upon donor-derived Ag re-challenge in the absence of any active immunosuppressive treatment), was 58% (Figure 1D). We accordingly conclude that the combination of rapamycin+IL-10 treatment with anti-CD45RB mAb promotes long-term tolerance in a stringent mouse model of islet transplant.

### Engraftment of allogeneic islets endorsed by the combined protocol is associated with CD4^+^CD25^+^Foxp3^+^ and CD4^+^IL-10^+^IL-4^−^ cells, which localize in different tissues

**Figure 2:**
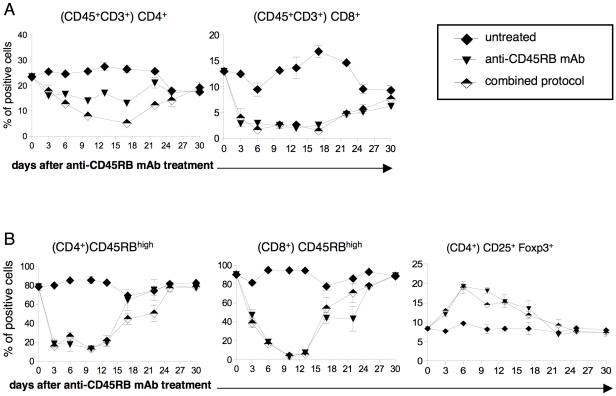
Anti-CD45RB mAb+rapamycin+IL-10 reduce CD45RB^high^ T cells and enrich CD4^+^CD25^+^Foxp3^+^ T cells. Diabetic B6 mice were transplanted with BALB islets and were either left untreated (n = 3), or treated with anti-CD45RB mAb (n = 4), or with the combined protocol (n = 5). (**A**) The frequencies of peripheral-blood CD4^+^ (left) and CD8^+^ (right) T cells within CD45^+^CD3^+^ cells are shown at different time points after the first injection of the anti-CD45RB mAb. (**B**) The frequencies of peripheral-blood CD45RB^high^ effector T cells within CD45^+^CD3^+^CD4^+^ cells (left) and within CD45^+^CD3^+^CD8^+^ cells (middle), and the frequencies of peripheral-blood CD25^+^Foxp3^+^ T cells within CD4^+^ cells (right), are shown at different time points after the first injection of anti-CD45RB mAb. One representative experiment out of 2 is shown. Means ± SEM are shown.

Our next step was to investigate the immunological outcome of the joint administration of anti-CD45RB mAb and rapamycin+IL-10. Kinetic analysis of the peripheral blood of transplanted and treated mice revealed that the number (data not shown) and the frequency of circulating CD4^+^ and CD8^+^ T cells was reduced (Figure 2A) as a result of specific CD45RB^high^ T-cell targeting by the anti-CD45RB mAb (Figure 2B), both in mice treated with the combined protocol and in those treated with anti-CD45RB mAb alone. In addition, the selective depletion of CD45RB^high^ cells led to an enrichment of circulating CD4^+^CD25^+^Foxp3^+^ Treg cells (Figure 2B). All these immunological phenomena were transient, since CD4^+^, CD8^+^, and CD4^+^CD25^+^Foxp3^+^ Treg cells returned to their physiological basal levels 30 days from the beginning of the treatment (Figure 2).

**Figure 3:**
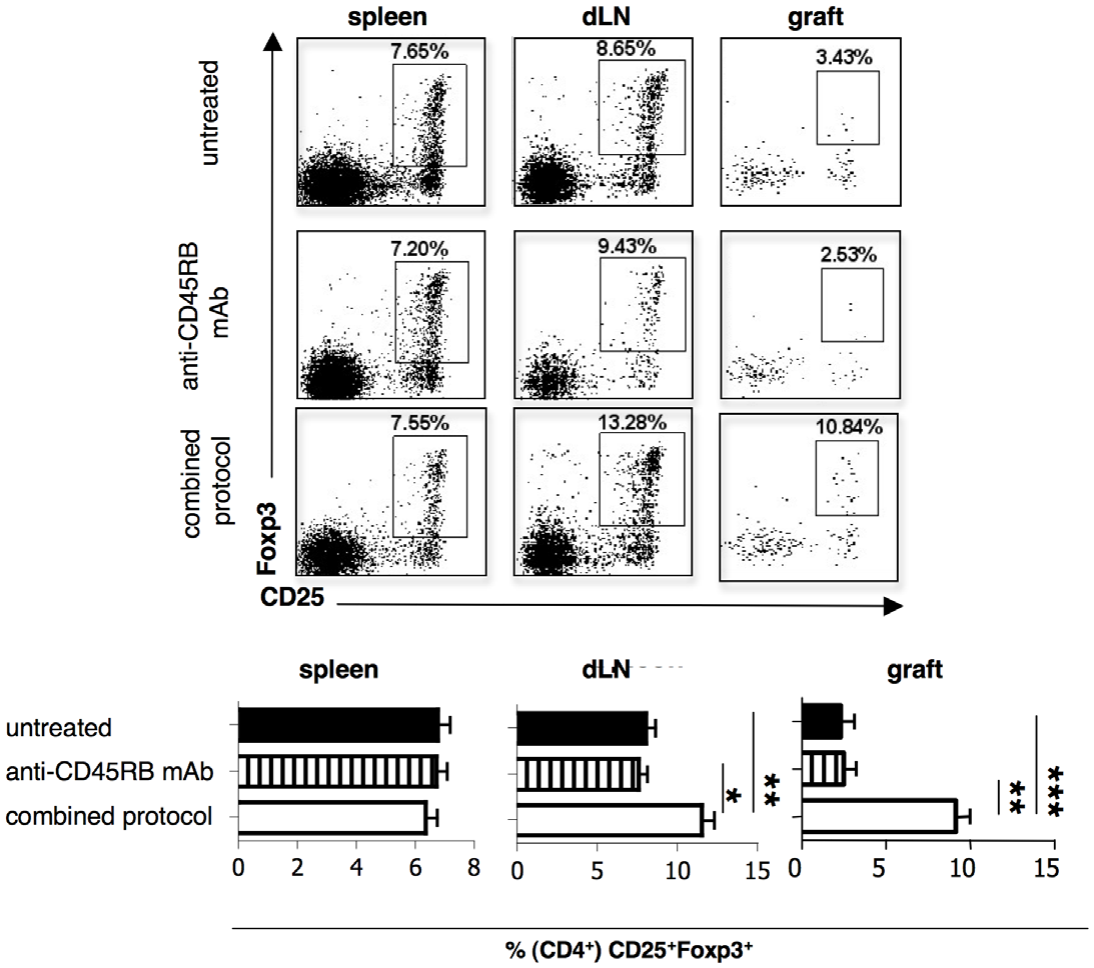
Localization of CD4^+^Foxp3^+^CD25^+^ Treg cells in engrafted mice after the combined protocol. Diabetic B6 mice were transplanted with BALB islets and were either left untreated (n = 13), or treated with anti-CD45RB mAb (n = 7), or with the combined protocol (n = 7). Thirty days after transplantation, treated mice were analyzed. Untreated control mice were analyzed once the graft was rejected. The frequencies of CD25^+^Foxp3^+^ cells within CD4^+^ cells in the spleen, in dLN, and in the graft are shown. Representative dot plots of the stainings are shown in the upper panel, while the results collected from all mice analyzed are shown in the lower panel. Means ± SEM are shown (*p<0.05;**p<0.005; ***p<0.0005).

**Figure 4:**
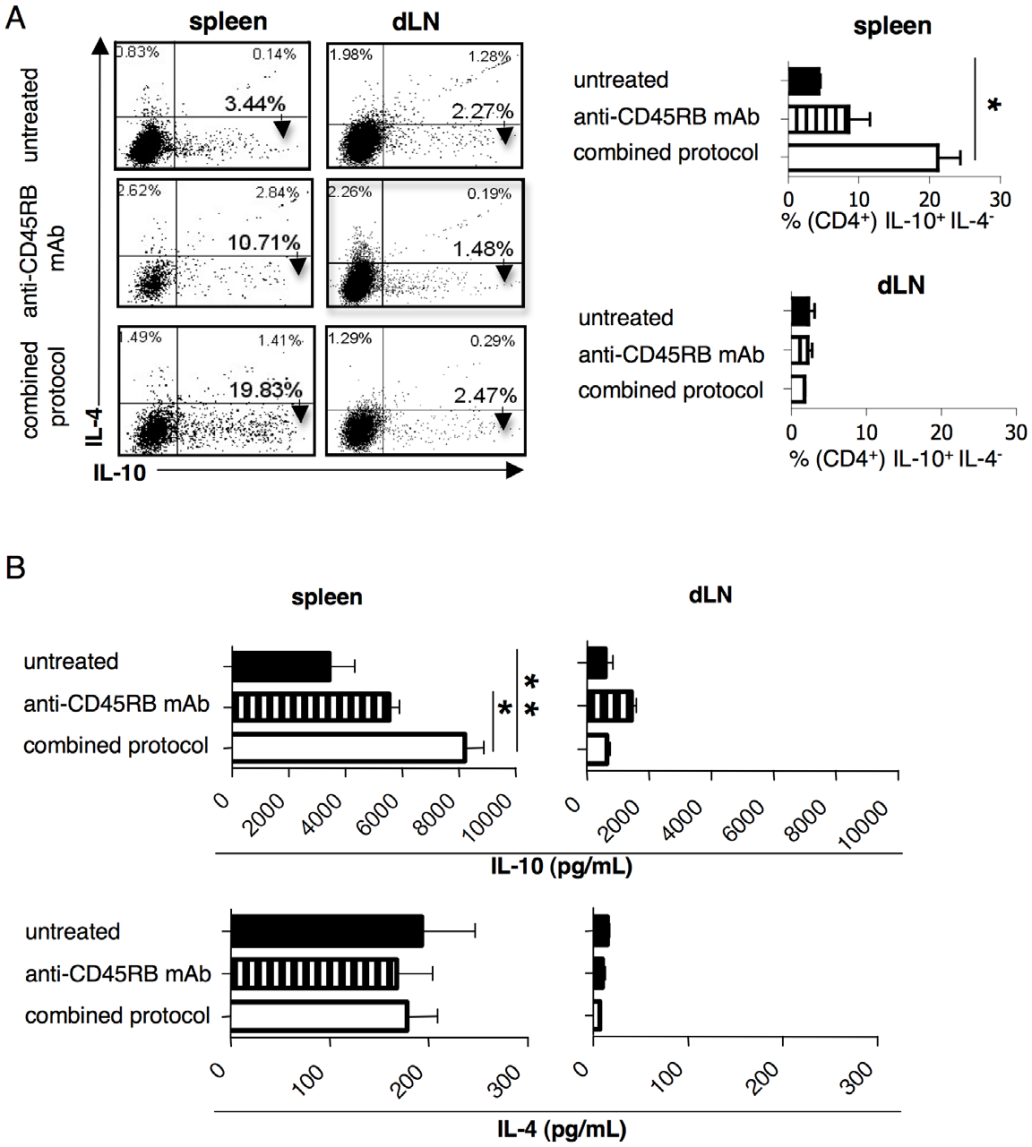
Localization of CD4^+^Foxp3^+^CD25^+^ Treg cells and ag-specific CD4^+^IL-4^−^IL-10^+^ T cells in engrafted mice after the combined protocol. Diabetic B6 mice were transplanted with BALB islets and were either left untreated (n = 13), or treated with anti-CD45RB mAb (n = 7), or with the combined protocol (n = 7). Thirty days after transplantation, treated mice were analyzed. Untreated control mice were analyzed once the graft was rejected. (**A**) Representative dot plots of the intracellular stainings for the detection of IL-10 and IL-4 production by CD4^+^ T cells from the spleens and the dLN are shown in the left panel, while the results collected from all mice analyzed are shown in the right panel. (**B**) The amounts of IL-10 (upper) and IL-4 (lower) released *in vitro* after polyclonal stimulation of CD4^+^ T cells isolated from the spleen (left) or the dLN (right) are shown. Cells isolated from spleen and dLN were cultured for 5 days in the presence of αCD3 (10 ug/ml) and αCD28 (1 ug/ml) mAbs. At the end of the culture the cells were finally re-stimulated for 5 h in the presence of TPA/ionomycin to test the ability to produce IL-10 and IL-4 was tested by intracellular staining. Means ± SEM are shown (*p<0.05;**p<0.005).

Given the known activity of rapamycin on Foxp3^+^ Treg cells, and of IL-10 on Tr1 cells [26], we analyzed these two Treg-cell types in the spleen, in the lymph-node that drain the graft (dLN), and in the graft of treated mice at the end of the treatment (*i.e.,* 30 days following transplantation). These analyses showed that the frequency of Foxp3^+^ Treg cells increased both in dLN and in the graft, but not in the spleen of mice treated with the combined protocol (Figure 3). In contrast, CD4^+^IL-10^+^IL4^−^ (*i.e.,* Tr1 cells) cells were enriched in the spleen, but not in the dLN nor in the graft (Figure 4A and data not shown). Accordingly, splenic CD4^+^ T cells isolated from mice treated with the combined protocol produced more IL-10 than did those isolated from control mice (Figure 4B), where the former also exert a powerful *in vivo* Ag-specific suppressive function, as we previously showed [12]. Interestingly, although treatment with anti-CD45RB mAb alone was not efficient in controlling graft rejection, it led to increased frequency of splenic CD4^+^IL-10^+^IL4^−^ T cells (Figure 4A). Consequently, the CD4^+^ T cells isolated from the spleen of the same treated mice produced more IL-10 than did those isolated from the spleen of control mice (Figure 4B).

Notably, no differences in the total number of CD4^+^ T cells were observed among untreated and treated (*i.e.,* anti-CD45RB mAb and combined) mice in the spleen, dLN and graft 30 days (Figure S1) and 150 days (data not show) following islet transplant. On the base of this, the frequencies of Foxp3^+^ Treg and Tr1 cells mirrored the differences in absolute number. Overall, these data show that the combined treatment promotes both the expansion of Foxp3^+^ Treg cells in the graft and dLN, and the induction of CD4^+^IL-10^+^IL4^−^ T cells that accumulate in the spleen.

### Long-term tolerance to allogeneic transplanted islets endorsed by the combined protocol is associated with splenic CD4^+^IL-10^+^IL-4^−^ cells

**Figure 5:**
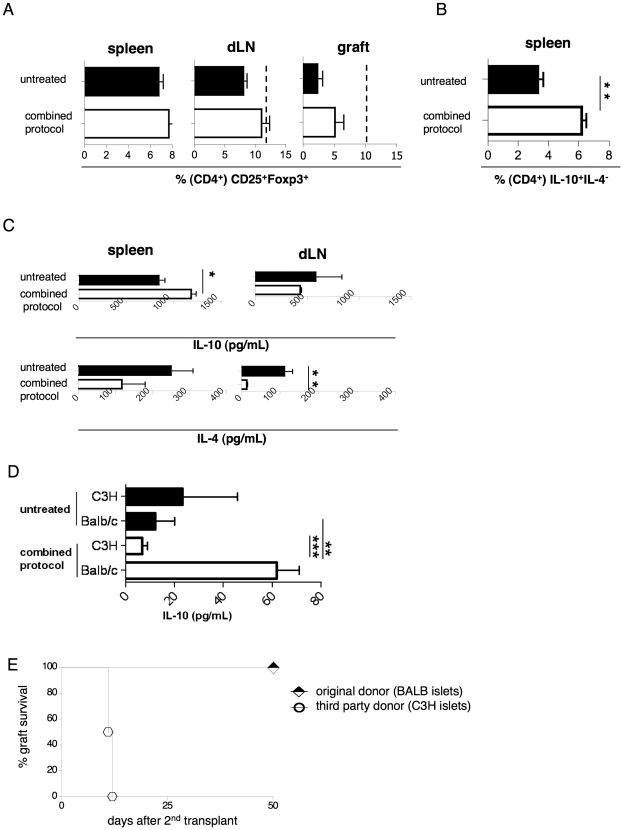
Ag-specific CD4^+^IL-4^−^IL-10^+^ T cells remain expanded in the spleen of tolerant mice and are associated with Ag-specific tolerance. Diabetic B6 mice were transplanted with BALB islets and were either left untreated (n = 8), or treated with the combined protocol (n = 5). Fifty days after boosting with donor-derived Ag (i.e., 150 days from transplantation), treated mice were analyzed. Untreated control mice were analyzed once the graft was rejected. (**A**) The frequencies of CD25^+^Foxp3^+^ cells within CD4^+^ cells in the spleen, in dLN, and in the graft are shown. The dash bars report the frequencies of CD25^+^Foxp3^+^ cells within CD4^+^ cells of transplanted mice treated with the combined protocol and sacrificed 30 days after transplantation. (**B**) The frequencies of IL-10^+^IL-4^−^ cells within splenic CD4^+^ cells were detected by intracellular staining, and **t**he results collected from all mice analyzed are shown. (**C**) The amounts of IL-10 (upper) and IL-4 (lower) released *in vitro* after polyclonal stimulation of CD4^+^ T cells isolated from the spleen (left) or the dLN (right) are shown. (**D**) The amounts of IL-10 released from the splenic CD4^+^ T cells *in vitro* after stimulation with third party Ag (C3H) or donor-specific Ag (BALB) are shown. Means ± SEM are shown (*p<0.05;**p<0.005; ***p<0.0005). (E) Diabetic B6 mice were transplanted with islets from BALB mice and were treated with the combined protocol. One hundred days after transplantation, the graft was removed and all the mice returned to being hyperglycemic. A second transplant was performed with islets isolated either from the original donor (BALB, n = 5) or from a third party donor (C3H, n = 4). The percentages of graft survival following the second islet transplant are shown.

To determine whether long-term tolerance (*i.e*., >150 days following transplantation) observed in mice treated with the combined protocol was still associated with the presence of Treg cells, we analyzed the frequency of Foxp3^+^ and CD4^+^IL-10^+^IL4^−^ T cells in mice 50 days from donor-derived Ag re-challenge (*i.e.,* 150 days from transplantation). The Foxp3^+^ Treg cells in the dLN had expanded to levels similar to those in the dLN of mice analyzed 30 days from transplantation, although said levels were not statistically significant in comparison to levels identified in untreated mice (Figure 5A). In the graft, the frequency of Foxp3^+^ Treg cells did not differ statistically from that found in untreated mice and was lower than that found 30 days following transplantation (Figure 5A). The lack of splenic accumulation of Foxp3^+^ Treg cell was also confirmed at this later time point (Figure 5A). The CD4^+^IL-10^+^IL4^−^ T cells remained enriched in the spleens of long-term tolerant mice (Figure 5B), and splenic CD4^+^ T cells produced high levels of IL-10 upon *in vitro* polyclonal stimulation (Figure 5C). In a demonstration of their donor-specificity, CD4^+^ T cells isolated from the spleen of tolerant mice produced IL-10 exclusively upon stimulation with original Ag (*i.e.,* BALB-derived splenocytes) and not with third party Ag (*i.e.,* C3H-derived splenocytes) (Figure 5D).

We then tested whether the presence of Ag-specific CD4^+^IL-10^+^IL4^−^ T cells was associated with Ag-specific long-term tolerance. The original graft was removed from long-term tolerant mice, in which diabetes promptly resumed, and a second transplant with islets isolated from the original donors or third-party donors was performed. Only mice receiving third party islets rejected the graft (Figure 5E), a finding that proved the establishment of Ag-specific long-term tolerance in mice transplanted with allogeneic islets and treated with the combined protocol.

### IL-10 is required for the maintenance of long-term tolerance

**Figure 6:**
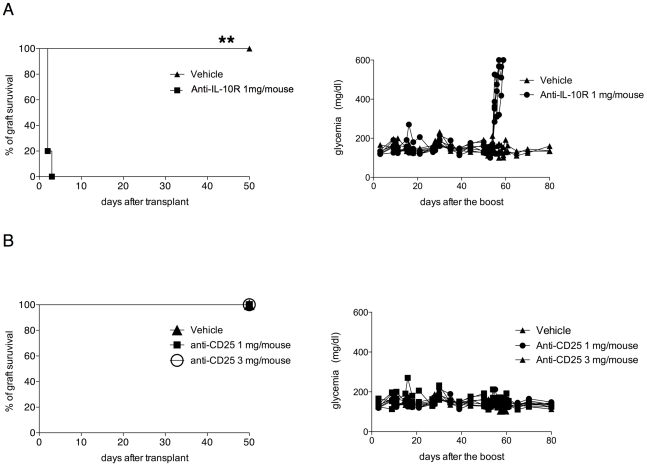
IL-10 and not CD4+ CD25+ Foxp3+ Treg cells is required to maintain long-term tolerance in transplanted mice upon the combined treatment. Diabetic B6 mice were transplanted with BALB islets and were treated with the combined protocol. (**A**) The percentage for graft survival (left) and the concentration of glycemia in the blood (right) of tolerant mice injected with the anti-IL-10R mAb (anti-IL-10R 1 mg/mouse) or with PBS (vehicle) at 150 days transplantation are shown. (**B**) The percentage for graft survival (left) and the concentration of glycemia in the blood (right) of tolerant mice injected with the low doe of anti-CD25 mAb (anti-CD25 mAb 1 mg/mouse), high dose (anti-CD25 mAb 3 mg/mouse) or with PBS (vehicle) at 150 days after transplantation are shown.

The combined protocol promotes a state of long term-tolerance associated with the accumulation IL-10-producing splenic CD4^+^ T cells. However, the role of IL-10 in this state of tolerance remains unknown. In order to address this point, tolerant mice were injected with anti-IL-10R mAb. Interestingly all the mice promptly rejected the graft upon mAb injection (Figure 6A).

Although the number and the frequencies of Foxp3^+^ T cells in tolerant mice were not significantly higher as compared to those in control mice, the combined treatment could have boost the regulatory activity of Foxp3^+^ T cells, for instance by increasing the expression of CTLA-4 [20]. Therefore, one could still hypothesize a role of these type of Treg cells in the maintenance of tolerance. Interestingly, the depletion of Foxp3^+^ T cells mediated by the injection of anti-CD25 mAb (Figure S2) did not alter the state of tolerance induced by the combined treatment (Figure 6B). These data show the key role of IL-10 in the maintenance of tolerance and suggest that Foxp3^+^ T cells are not the IL-10-producers.

### A clinically translatable protocol (G-CSF+rapamycin) induces long-term tolerance

**Figure 7:**
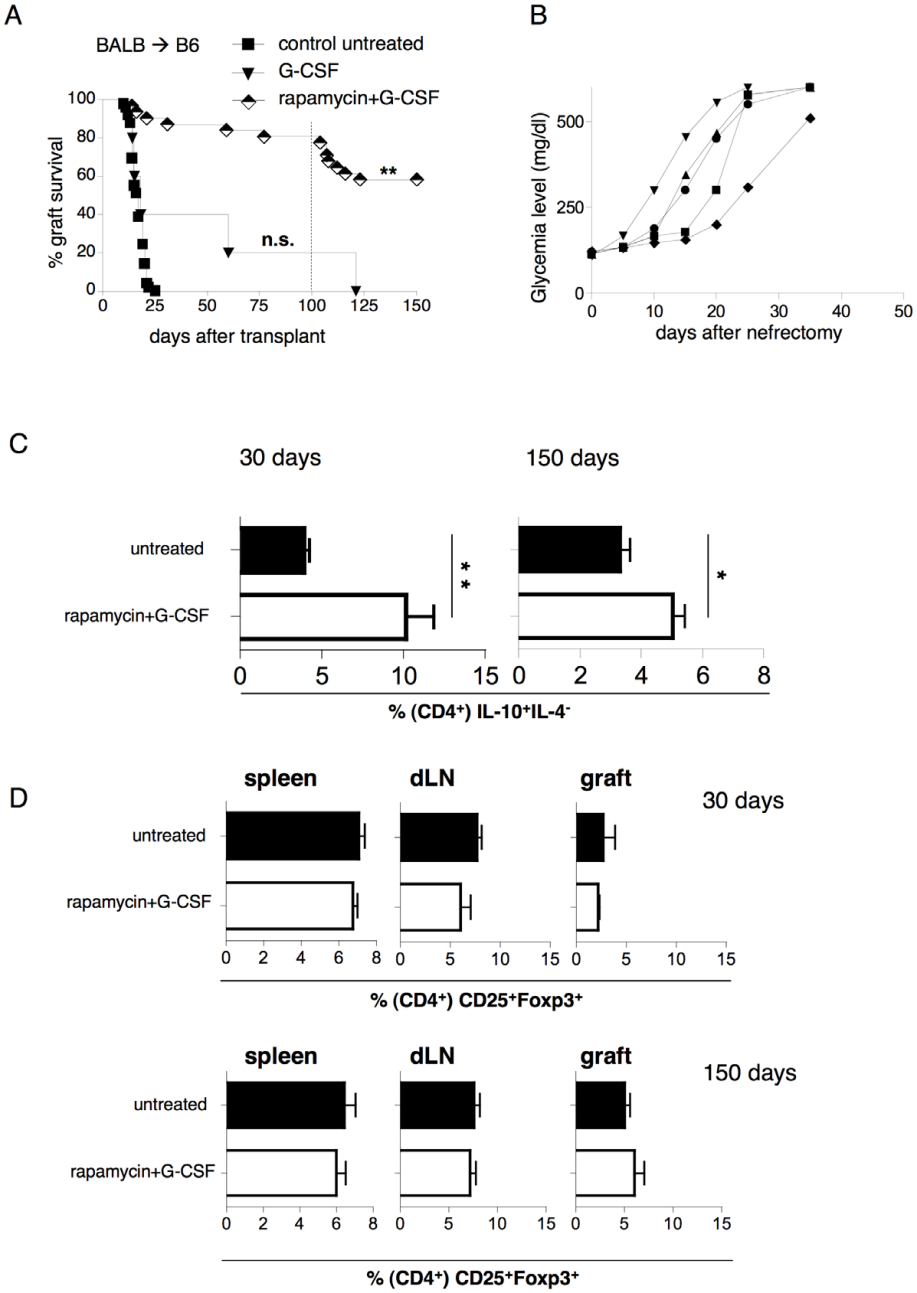
G-CSF+rapamycin induces long-term tolerance associated with splenic CD4^+^IL-4^−^IL-10^+^ T cells. Diabetic B6 mice were transplanted with BALB islets. (**A**) The transplanted mice were either left untreated (n = 47) or treated with G-CSF alone (n = 5) or with rapamycin+G-CSF (n = 30). The treatment was withdrawn 30 days after transplantation. One hundred days after transplantation, donor-derived splenocytes were injected into tolerant mice to boost their immune system. Overall graft survival is shown. These results are cumulative of 2 independent experiments performed in parallel. Log-rank (Mantel-Cox) test has been used (*p<0.01;**p<0.001;) (**B**) The graft was surgically removed from tolerant mice (n = 5) to verify graft functionality. Glycemia levels in the blood of each mouse tested are shown. (**C**) The frequencies of IL-10^+^IL-4^−^ T cells within splenic CD4^+^ T cells 30 (left) and 150 (right) days after transplantation in control untreated (n = 5) and rapamycin+G-CSF–treated (n = 5) mice are shown. Cells isolated from spleen and dLN were cultured for 5 days in the presence of αCD3 (10 ug/ml) and αCD28 (1 ug/ml) mAbs. At the end of the culture the cells were finally restimulated for 5 h in the presence of TPA/ionomycin to test the ability to produce IL-10 and IL-4 was tested by intracellular staining. (**D**) The frequencies of CD25^+^Foxp3^+^ T cells within CD4^+^ cells in the spleen, in dLN and in graft 30 (upper) and 150 days (lower) after transplantation in control untreated (n = 5) and rapamycin+G-CSF–treated (n = 5) mice are shown. Means ± SEM are shown (*p<0.05;**p<0.005).

One important goal in the field of experimental (*i.e.,* preclinical) transplantation is to develop an immunomodulatory protocol that is readily translatable to the clinic setting. Since recombinant IL-10 is not available for clinical use, we chose to replace it with G-CSF. G-CSF is a growth factor currently used for the clinical mobilization of hematopoietic stem cells [27]. Importantly, both in mice [21] and in humans [28], G-CSF has tolerogenic properties that resemble those of IL-10. The combination of G-CSF+rapamycin, in the absence of any depleting agents, led to long-term graft survival in 80% of B6 mice transplanted with BALB islets (*i.e.,* stringent model) (Figure 7A). Upon injection of donor-derived splenocytes (*i.e.,* donor-derived antigen boost) 100 days from transplant, only 7 out of 25 mice with a long-term functioning graft rejected the allogeneic islets. Overall, 58% of mice treated with G-CSF+rapamycin achieved long-term tolerance (Figure 7A). Finally, graft removal from all tolerant mice promptly induced a return to hyperglycemia in each animal, and thus confirmed the lack of any endogenous islet regeneration (Figure 7B).

Treated mice were analyzed, and increased percentages of CD4^+^IL-10^+^IL4^−^ T cells were found in the spleen 30 and 150 days from transplantation (Figure 7C). In contrast, Foxp3^+^ Treg cells were not found expanded in the spleen, dLN, or in the cells infiltrating the graft; these findings were confirmed both at the end of the treatment and in long-term tolerant mice (Figure 7D).

These data demonstrate that treatment with G-CSF+rapamycin endorses long-term tolerance in a stringent mouse model of islet transplantation, without any need for a deletion compound. Such transplant tolerance is associated with the induction of splenic CD4^+^ with a Tr1-cell phenotype. However, further studies are required to demonstrate the role of Tr1 cells for the maintenance of tolerance upon G-CSF+rapamycin treatment.

## Discussion

Firstly, we have proved that the combination of rapamycin, IL-10 and anti-CD45RRB mAb is an efficient protocol to induce Ag-specific tolerance as associated with two different types of Treg cells localized in different tissues. Interestingly, the engraftment was accompanied by the accumulation of Foxp3^+^ Treg cells in the target organ (*i.e.,* graft) and of Ag-specific CD4^+^IL-10^+^IL-4^−^ T cells in the spleen. In contrast, long-term donor-specific transplantation tolerance is characterized by a small collection of Foxp3^+^ Treg cells in dLN and by the maintenance of Ag-specific CD4^+^ IL-10^+^ IL-4^−^ T cells in the spleen. Accordingly the tolerance is maintained by IL-10, while Foxp3^+^ Treg cells are dispensable. Secondly, we have shown that the combination of rapamycin with G-CSF, two drugs already in clinical practices, promotes long-term tolerance in the stringent mouse model of islet transplantation. It is interesting to note that this tolerance was again associated with the presence of CD4^+^IL-10^+^ IL-4^−^ T cells in the spleen of treated mice. However, the role of IL-10 and in turn of Tr1 cells remains to be investigated.

Previous studies had already shown the transient deletion of circulating lymphocytes following treatment with anti-CD45RB mAb [18], [19]. We have confirmed this phenomenon and demonstrated that Foxp3^+^ Treg cells are spared, while naïve/effector CD4^+^, CD8^+^ T cells and B cells (data not shown) are the main targets. The injection of anti-CD45RB mAb promotes Foxp3^+^ Treg-cell enrichment in the blood since the expression of CD45RB by Foxp3^+^ Treg cells is low [29]. The frequency of Foxp3^+^ Treg cells returns to normal levels upon the repopulation of the blood with naive/effector CD4^+^ and CD8^+^ T cells. This paper shows that these effects are maintained when anti-CD45RB mAb is combined with rapamycin+IL-10 treatment.

It is also known that the anti-CD45RB mAb fits the regulatory properties of Foxp3^+^ Treg cells by increasing the expression of CTLA-4 and by promoting long-term graft survival [20]. We confirm that increased CTLA-4 expression coincides with high engraftment efficacy in anti-CD45RB mAb-treated mice. However, none of these treated animals reached a state of long-term tolerance. This result partially contrasts with previously published data [30]. We suggest that these varying results could be explained by: (I) differences in administration schedules; (II) differences in gender ratios of the mice used as recipients and donors; and (III) differences in approach used to verify the state of tolerance (*e.g.,* boost with splenocytes versus a second transplantation). Importantly, we have clearly demonstrated that anti-CD45RB mAb radically increases the efficacy of rapamycin+IL-10 treatment in the stringent mouse model of islet transplantation.

Our data demonstrate that after the combined treatment, Foxp3^+^ Treg cells and CD4^+^IL-10^+^IL-4^−^ T cells localized in different organs. Data from mice and also from humans have already reported that Foxp3^+^ Treg cells occur in tolerant grafts [31]. Other authors suggest that the presence of Foxp3^+^ Treg cells in dLN are key for their regulatory action [31]. In our model, we observed Foxp3^+^-Treg cells in the graft and in dLN 30 days from transplantation, and their subsequent accumulation in the dLN of long-term tolerant mice. This suggests that Foxp3^+^ Treg cells may migrate from the graft to dLN. In agreement with our hypothesis, this Foxp3^+^ Treg-cell migration (*i.e.,* from the graft to dLN) has been found to be a requisite for the blocking of islet allograft rejection [32]. Interestingly, our data suggest that at later time points, once immunological tolerance is established, the Foxp3^+^ Treg cells slightly accumulate in the dLN and they are dispensable for the maintenance of tolerance. Accordingly, studies performed in mouse models of islet transplant have demonstrated that Foxp3^+^ Treg cells are fundamental only during the early phase after transplantation [33], [34], [35]. On the basis of this, one could hypothesizes that Foxp3^+^ Treg cells migrate to the graft at early time points, exert their main role *in situ*, and become dispensable lately once localized in the dLN.

Unlike the localization of Foxp3^+^ Treg cells, Tr1 cells localize mainly in the spleen, as we previously observed in various animal models of tolerance [17], [26] and as we reassert, with increased evidence, in this study. It is especially interesting to note that splenic DCs alone have the capacity, in the presence of TGF-β, to induce Tr1 cells [36]. However, the issue of whether the spleen is the key organ for Tr1-cell regulatory activity is still inadequately addressed.

All in all, the combined treatment promoted a state of tolerance that was associated with two different types of Treg cells, which in turn localized in differing tissues. This model represents a unique opportunity to study some still ill-defined aspects of the biology of Foxp3^+^ Treg and Tr1 cells: whether, where and how these two different types of Treg cells work together to induce and promote transplantation tolerance.

Exogenous treatment with recombinant IL-10 has not clearly demonstrated beneficial effects in a number of clinical trials [37], and at present is not clinically available. On this basis, we developed a new combined protocol that uses previously approved compounds. G-CSF is used for the mobilization of stem cells, and it shows tolerogenic capacities that are interestingly similar to those of the IL-10 treatment [27]. We accordingly substituted IL-10 with G-CSF; without using any depleting agents, and against all expectation, the combination of rapamycin+G-CSF promoted tolerance in the stringent mouse model of islet transplantation. Although it is known that G-CSF promotes the in vivo expansion of CD4^+^CD25^+^ T cells [22], [23], we could not find any enrichment of these cells in any target organs of treated mice. Differences in G-CSF administration schedules and/or the combination with rapamycin could explain why our data vary from previous findings.

It is also known that G-CSF promotes the release of IL-10 from human and mouse CD4^+^ T cells [21], [28]. In turn, we find a strong in vivo expansion of splenic CD4^+^ IL-10^+^ IL-4^−^ T cells, both under the umbrella of the treatment and in tolerant mice. Although the cytokine profile of CD4^+^ IL-10^+^ IL-4^−^ T cells found in the spleen of treated mice reflects that of Tr1 cells, future efforts must verify their regulatory capacity. We are currently developing new tools that will selectively isolate or delete Tr1 cells as a way to understand their specific role in the induction of tolerance. Finally, it is interesting to note that two different treatments induce CD4^+^ IL-10^+^ IL-4^−^ T cells, which in both cases localize in the spleen and occur in tolerant mice.

## Materials and Methods

### Mice and islet transplant

BALB/c (BALB) and C57BL/6 (B6) female mice were purchased from Charles River (Calco, Italy). All mice were maintained under specific pathogen-free conditions. Diabetes was induced by intravenous (*i.v.*) injection of 170 mg/kg streptozotocin (Sigma-Aldrich, St. Louis, MO). Glucose levels in tail venous blood were quantified with the Glucometer Elite system (Bayer, Wuppertal, Germany), and measurements invariably took place in the morning. Pancreatic islet transplant was performed as previously described [17]. Graft rejection was diagnosed on the basis of two sequential glucose measurements >300 mg/dl.

The transplanted mice that did not reject the graft 100 days after transplantation were boosted *in vivo* with donor-origin splenocytes. A total of 30×10^6^ splenocytes isolated from the original islet donors were injected intraperitoneally (*i.p.*), and blood glucose levels were monitored daily thereafter. Mice still normoglycemic 30–50 days from boosting were considered long-term tolerant. The study was approved by the San Raffaele Hospital Institutional Animal Care and Use Committee (permit no IACUC 350).

### Treatment of transplanted mice

Transplanted mice were treated with anti-CD45RB mAb (MB23G2 clone from ATCC) alone or with the addition of rapamycin (Rapamune; Wyeth Europe, Taplow, U.K.)+r-hu-IL-10 (BD Pharmingen, San Diego, CA). Anti-CD45RB mAb was injected *i.v.* at days 0, 1, and 5 following transplantation at 100 µg per dose. Rapamycin was diluted in water and administered by gavage for 30 consecutive days once a day at 1 mg/kg. Recombinant hu-IL-10 was diluted in PBS and administered *i.p.* twice a day for 30 consecutive days at 0.05 µg/kg. G-CSF (Myelostim, rHuG-CSF, Italfarmaco) was diluted in PBS and injected *s.c.* for 30 consecutive days at 200 µg/kg. Neither treatments with the vehicles (water, PBS), nor with the isotype control for anti-CD45RB mAb (Rat IgG2a), following the same administration and schedule of the original treatments, show any delay in the survival of the graft respect to untreated mice (data not shown).

Some tolerant mice (combined treatment) were injected either with the anti-IL-10 receptor (anti-IL-10R) (1B1.2 clone from ATCC, Manassas, VA) or with the anti-CD25Rα (PC61 clone from ATCC) monoclonal antibody (mAb). The anti-IL-10R mAb diluted in PBS (vehicle) was administered *i.p.* for three consecutive days to reach a dose of 1 mg/mouse. The PC61 mouse (500 mg/mouse/dose) was diluted in PBS (vehicle) and administered *i.p.* for two consecutive days every week for either 1 or 3 weeks to reach the dose of 1 mg/mouse and 3 mg/mouse respectively. The doses of anti-IL-10R and PC61 mAbs were chosen in accordance with the literature [17], [33], [34].

### Cell isolation

Spleen, dLN and graft were isolated from transplanted mice and mashed to yield single cell suspensions. Splenic CD4^+^ T cells were magnetically isolated in accordance with the protocol of the “CD4^+^ T cell isolation kit” (Miltenyi Biotec, Bergisch Gladbach, German; purity 90–95%).

### Cell staining and enzyme-linked immunosorbent assay

Surface cell staining was performed with anti-CD45 PercP, anti-CD3 APC, anti-CD4 Pacific Blue, anti-CD25 APC or PeCy7, and anti-CD8 PE mAbs (all from BD Pharmingen). The CD45RB expression was tested with the anti-CD45RB PE mAb (clone 16A from BD Pharmingen), which had previously been demonstrated to bind to an epitope different than that bound by MB23G2 mAb [20]. Foxp3 expression was tested with the Foxp3 staining kit (eBioscience, San Diego, California; clone FjK-16s). Intracellular staining and ELISA were performed as previously described [12]. Cells were acquired on a FACSCanto (BD Bioscience) and analyzed with FCS express V3 (De Novo Software, Los Angeles, CA).

### Statistical analysis

Differences between groups were assessed with the Student t test. P values were two-tailed with a confidence of 95%. Islet allograft survivals were determined with Kaplan-Meier survival curves, and were compared by means of the log-rank test. Statistical analysis was performed with the Prism V4.03 software (Graph-Pad, San Diego, California USA).

## Supporting Information

Figure S1 **Diabetic B6 mice were transplanted with BALB islets and were either left untreated (Untreated), or treated with anti-CD45RB mAb (aCD45RB), combined protocol (Combine), G-CSF (C-CSF) and rapamycin plus G-CSF (rapamycin+G-CSF).** Thirty days after transplantation, treated mice were analyzed. Untreated control mice were analyzed once the graft was rejected. The total number of CD4+ T cells within CD45+ cells in the spleen, in dLN, and in the graft is shown. Results are cumulative from three independent experiments. The study was approved by the San Raffaele Hospital Institutional Animal Care and Use Committee (permit no IACUC 350).
(TIF)

Figure S2 **B6 mice were injected i.v. with 1 mg (2 injections) or with 3 mg (6 injections) of anti-CD25 mAb (clone PC61) and 7 days after the last injection the % of CD25+ Foxp3+ within CD4+ T cells in the blood, spleen and mLN were analyzed.** The clone 7D4 for mAb anti-CD25 has been used for the FACS staining. The study was approved by the San Raffaele Hospital Institutional Animal Care and Use Committee (permit no IACUC 350).
(TIF)
