# Supplementary figures and images for: Rapamycin Combined with Anti-CD45RB mAb and IL-10 or with G-CSF Induces Tolerance in a Stringent Mouse Model of Islet Transplantation
Source: PLoS One. 2011 Dec 9;6(12):e28434. doi: 10.1371/journal.pone.0028434 (PMC3235119; doi:10.1371/journal.pone.0028434)

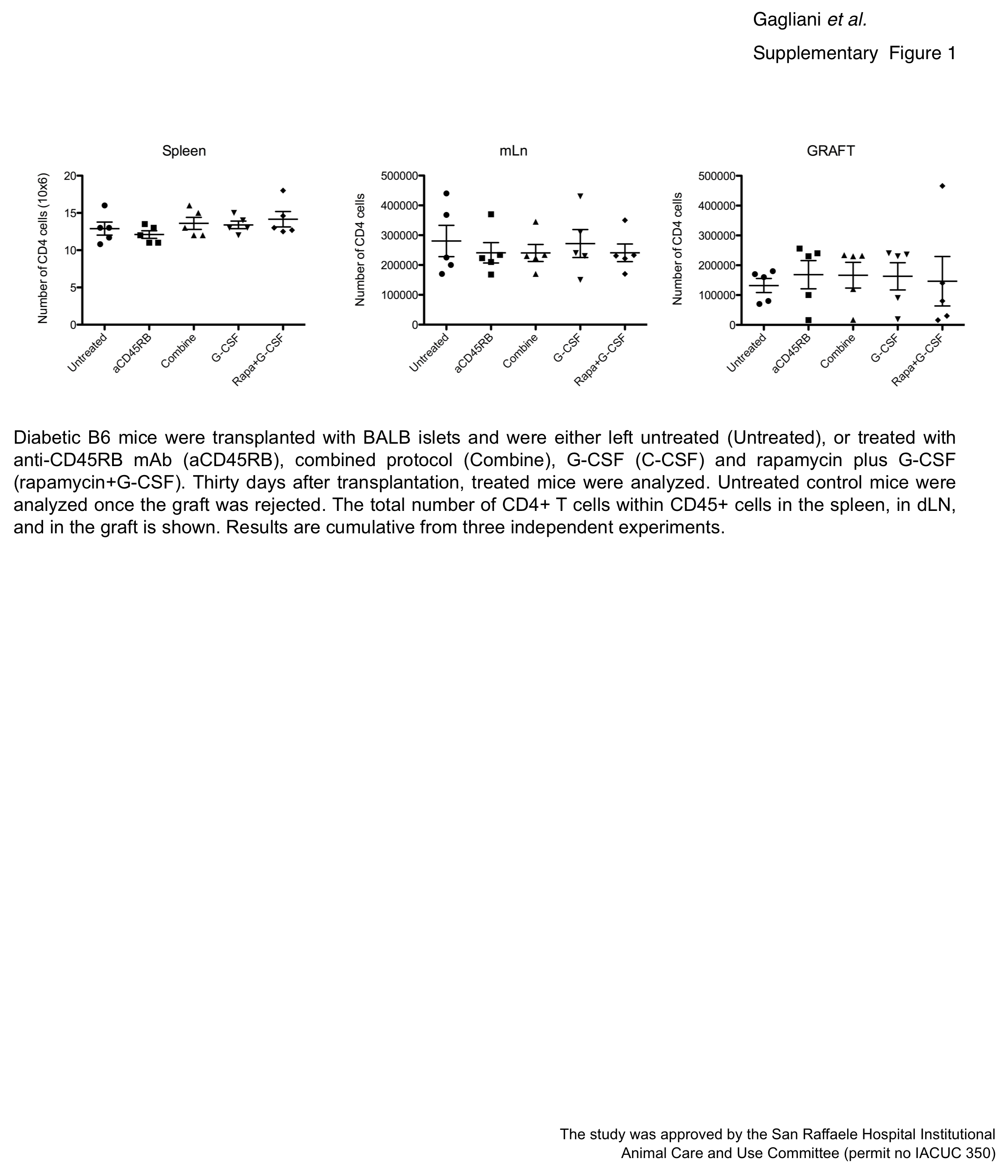

Supplement: Figure S1 — Diabetic B6 mice were transplanted with BALB islets and were either left untreated (Untreated), or treated with anti-CD45RB mAb (aCD45RB), combined protocol (Combine), G-CSF (C-CSF) and rapamycin plus G-CSF (rapamycin+G-CSF). Thirty days after transplantation, treated mice were analyzed. Untreated control mice were analyzed once the graft was rejected. The total number of CD4+ T cells within CD45+ cells in the spleen, in dLN, and in the graft is shown. Results are cumulative from three independent experiments. The study was approved by the San Raffaele Hospital Institutional Animal Care and Use Committee (permit no IACUC 350). (TIF) [file pone.0028434.s001.tif]

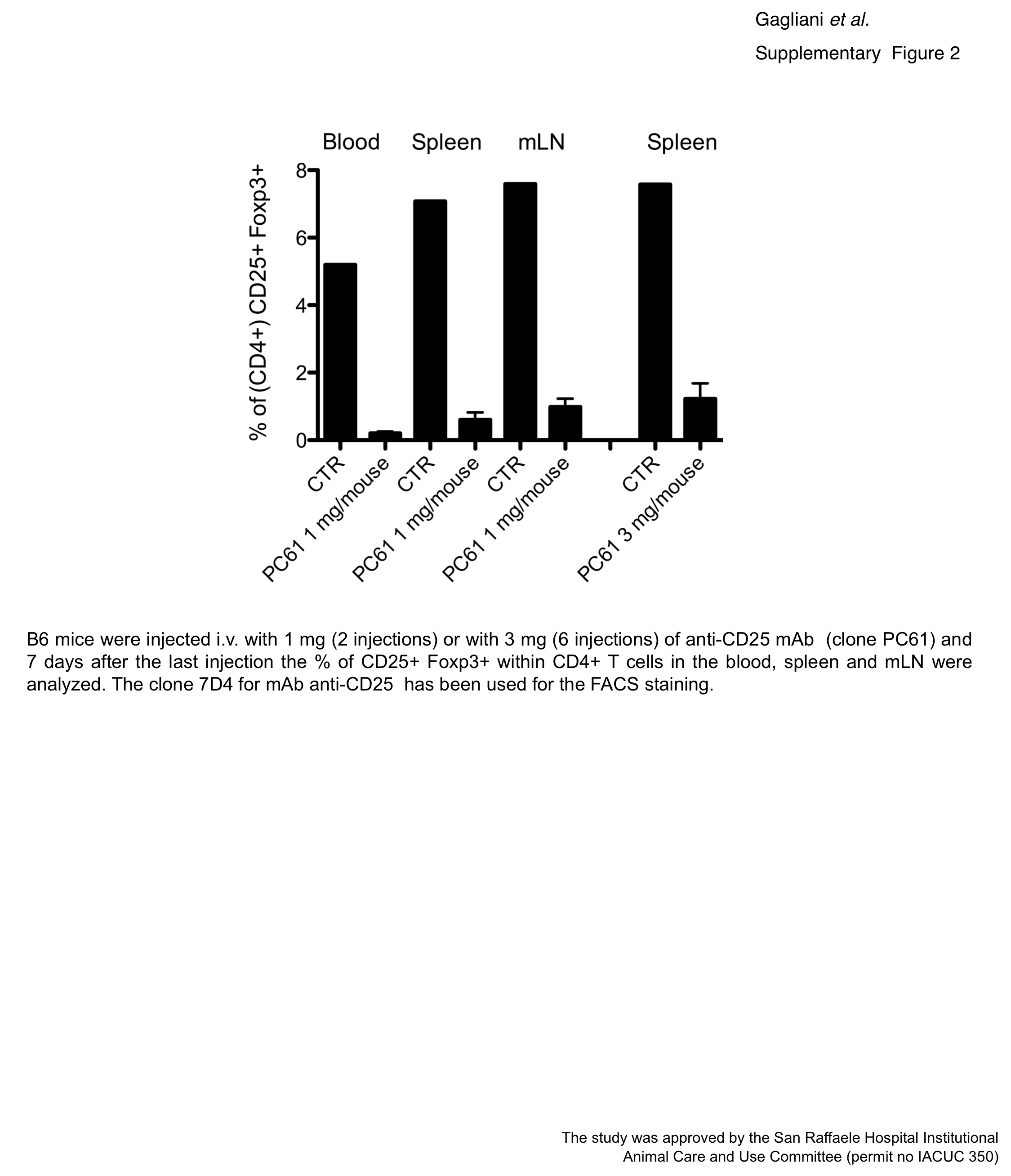

Supplement: Figure S2 — B6 mice were injected i.v. with 1 mg (2 injections) or with 3 mg (6 injections) of anti-CD25 mAb (clone PC61) and 7 days after the last injection the % of CD25+ Foxp3+ within CD4+ T cells in the blood, spleen and mLN were analyzed. The clone 7D4 for mAb anti-CD25 has been used for the FACS staining. The study was approved by the San Raffaele Hospital Institutional Animal Care and Use Committee (permit no IACUC 350). (TIF) [file pone.0028434.s002.tif]
